# Isolation and Characterization of Potential Antibiotic-Producing Actinomycetes from Water and Soil Sediments of Different Regions of Nepal

**DOI:** 10.1155/2021/5586165

**Published:** 2021-03-03

**Authors:** Bhanu Shrestha, Dharmendra Kumar Nath, Alina Maharjan, Anju Poudel, Roshani Nhuchhen Pradhan, Sagar Aryal

**Affiliations:** ^1^Department of Microbiology, St. Xavier's College, Maitighar, Kathmandu, Nepal; ^2^Department of Natural Products, Kathmandu Research Institute for Biological Science, Lalitpur, Nepal

## Abstract

**Introduction:**

The actinomycetes are diversely distributed microorganisms in nature. The geographical diversity of Nepal is suitable for adaptation of various species of actinomycetes. The distribution of the actinomycetes is dependent upon the altitude and nature of the soil and water.

**Methods:**

A total of 22 water and soil samples were collected from different regions of Nepal and were processed. Different isolates were characterized by observing colony characteristics and microscopic characteristics. Screening of the antimicrobial property was based upon perpendicular line streaking and submerged-state fermentation for antibiotic production.

**Results:**

From the identification tool used, 12 were found to be *Micromonospora*, 9 were *Nocardia,* and 7 were *Streptomyces.* Out of total samples, 8 isolates of actinomycetes were tested effective against the tested bacteria by primary screening using the well diffusion method. Among the primarily screened, all isolates were subjected to submerged-state fermentation methods to produce crude extracts and 4 were found to be effective against the tested bacterial group. The actinomycetes identified from a water source showed better antimicrobial property towards the tested bacteria than the soil sample. Most isolates were found to be *Micromonospora* followed by *Nocardia* and *Streptomyces* with higher antimicrobial activities.

**Conclusion:**

The water source and soil sediments of Nepal provide suitable environments for actinomycetes towards obtaining a novel antimicrobial agent. The study of actinomycetes from various unexploited areas of Nepal is necessary. Thus, exploitation of various regions of Nepal for the discovery of an effective antimicrobial agent is helpful in providing a solution to the cost-effective therapy and action against antibiotic resistance.

## 1. Introduction

Actinomycetes (derivation- Actini: ray, mycetes: fungi) are Gram-positive filamentous bacteria which are acid fast in nature. Though actinomycetes are recognized as Gram-positive bacteria, they differ from other bacteria having their morphological characteristics such as high guanine plus cytosine (*G* + *C*) contents in their DNA [1, 2]. Actinomycetes are the most widely distributed group of microorganisms in nature which can be found everywhere including soil and water sources. They have been very useful in antibiotic production naturally. Different antibiotics can be extracted from *Actinomycetes*, namely, tetracycline, macrolide, chloramphenicol, nucleosides, and polyenes. Many antibiotics, namely, erythromycin, gentamycin, rifamycin, and streptomycin, are the products derived from soil actinomycetes [3]. The availability of antibiotic-producing actinomycetes varied depending on the texture and cultivation status of the soil [4]. Although actinomycetes are present globally, only few have been screened [5]. Thus, there is a high probability of isolating variable actinomycetes that produce antibiotics from the water source as soil and water are comparatively related natural sources [6]. If the antibiotics can be extracted from the water source, it would reduce the effort, ultimately leading to reduction in price which can prove as a boon for most of the underprivileged people in developing nations like Nepal.

In case of biotechnological application, actinomycetes have been greatly studied and found to be the most useful bacteria. Actinomycetes are one of the major sources of antibiotics [7]. *Streptomyces* are the predominant members of actinomycetes which are discerned as a major bioactive compound producer [8]. Till date, among the 22,500 biologically active compounds obtained from microbes, 45% are from *Actinomycetes*, 38% are from fungi, and 17% are from other bacteria. Also, the species of *Streptomyces* only accounts about 70% of total antibiotics production [9]. The discovery of rare actino products in 1970 was only 5% which increased to 45% till date, and data are still increasing for the fulfillment of the demand.

The rate of discovery of new compounds from terrestrial actinomycetes has decreased, but the rate reisolation of known compounds has increased [10]. Discovery of new antibiotics from new actinomycetes groups should be stressed since only 1–10% of the total microbial flora on the earth has been properly studied [11]. In most of the classes of antibiotics, there is resistance developed in bacteria encompassing their use and in some antibiotics of the same class showing prompting cross resistance to that class of drugs [12]. Thus, it has been necessary to identify new groups of actinomycetes from poorly explored or unexplored habitats to be undertaken as sources of novel bioactive secondary metabolites [13]. The actinomycetes products are used for curable treatment, and despite production of useful secondary metabolites in large numbers, it seems to limit on the identification and production process. Also, the development of a resistant gene in bacteria has led urgent requirement of flourishing vegetation for possible antibiotics [14]. Thus, an immediate and full-phase study about the antimicrobial property of actinomycetes and antibiotics produced from them will be necessary in the near future.

Serious bacteria have been developed against commonly used antibiotics in the present scenario [15]. Rise of resistance of antibiotics developed is a leading major problem in the health sector. This has been threatening from the early discovery of penicillin in 1943 whose resistance *Pneumococcus* was emerged in 1965. Similarly, a recently discovered antibiotic, ceftaroline in 2010, also had a problem with the development of ceftaroline-resistant *Staphylococcus* in 2011 (source: CDC). A common bacteria *Staphylococcus aureus* has developed resistance to most class of antibiotics [16]. Thus, there are urgent requirements of development of new antimicrobial agents to solve these problems. Furthermore, Nepal, at the co-ordinate of 26° 22′ N to 30° 27′ N latitude and 80° 04′ *E* to 88° 12′ *E* longitude, is a narrow rectangular country in the Himalayas having different ranges of land topography, namely, Terai, Hilly, and Himalaya (mountain). The diverse topography leads to different textures and compositions of soil, as well as water source, leading to habitation of different microbial flora, among which actinomycetes also accounts for few percentages. The geography of soil location, rearing, and organic content play a great role in affecting the availability and diversity of the actinomycetes present [5]. The exploitation of these regions for the isolation of potential antimicrobial-producing bacteria including actinomycetes is found to be prior in the to-do list for the fulfillments of demand of novel antibiotics. Thus, the present study is intended to isolate, screen, and characterize the antibiotic-producing *Actinomycetes*, exploring the potential source of actinomycetes from different regions of Nepal.

## 2. Materials and Methods

### 2.1. Sampling and Sample Site

From the different altitude of Nepal, 17 water samples and 5 soil samples were collected in a bottle (125 ml bottle, #CLS8388, Sigma) aseptically from different regions varying in the altitude and geographical distribution. The collected samples were taken to the research laboratory of the Department of Microbiology, St. Xavier's College, Maitighar, Nepal, where the remaining complete processing were carried out. During the sample collection, the highest altitude for the sample site was 1853 meter while the lowest altitude was 968 meters from sea level. The pH measurement and texture evaluation of the sample were carried out immediately after the sample reached the laboratory.

### 2.2. Isolation of *Actinomycetes*

**Figure 1:**
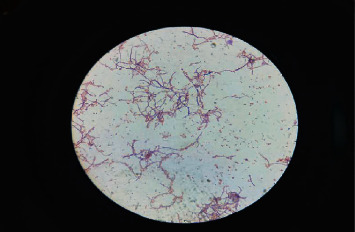
Microscopic observation of actinomycetes isolated. The microscopic observation of actinomycetes using the Gram staining method. Gram-positive red color rod-shaped bacteria with branch network of hyphae are observed under the microscope.

For the soil sample, 1 gram was weighed and mixed well in a test tube containing 10 ml of distilled water. It was then serially diluted up to 10^−5^ dilution. From the serial dilution of 10^−3^ and 10^−5^, 0.1 ml was spread plated on the Starch Casein Agar (SCA) plate (#95022–272), Himedia (composition: soluble starch: 10 g, K_2_HPO_4_: 2 g, KNO_3_: 2 g, casein: 0.3 g, MgSO_4_.7H_2_O: 0.05 g, CaCO_3_: 0.02 g, FeSO_4_.7H_2_O: 0.01 g, agar: 15 g, and filtered sea water: 1000 ml and pH: 7.0 ± 0.1). The plates were incubated for 4–5 days at 28°C for isolating the colony of actinomycetes. Repeated subculture was performed by streaking on the SCA to obtain pure culture [16]. Different color isolated colonies with hard texture and powdery and musty form were identified based upon the macroscopic observation which were distinct from another bacterial colony. Furthermore, the microscopic observation was performed using Gram staining for observation of the thin thread-like mycelial and hyphal form [6] (Figure 1).

### 2.3. Bacteria Strain Used

Different pathogenic Gram-positive and Gram-negative bacteria such as *Escherichia coli* (ATCC 25922)*, Staphylococcus aureus* (ATCC 25923), *Klebsiella pneumoniae* (ATCC 700603), *Bacillus* spp., and *Pseudomonas aeruginosa* (ATCC 27853) were used as the test bacteria in the primary screening.

### 2.4. Primary Screening of the Isolates

After the proper subculture, subcultured isolates were used to streak lines on the Mueller Hinton Agar (MHA) (#70191, Sigma, composition: beef infusion solids- 2gm/l, starch- 1.5gm/l, casein hydrolysate- 17.5gm/l, and agar- 17gm/l; final pH 7.3 ± 0.2 at 25°C) which was incubated for 2–3 days on 28°C. During the primary screening, these streaked lines of actinomycetes were screened against the abovementioned pathogenic bacteria by drawing a perpendicular line to the previous line of actinomycetes. Results showing the zone of inhibition were observed within 24 hours after proper incubation at 37°C, and the zone of inhibition was recorded [4].

### 2.5. Production of Crude Extract

The isolates showing an antibiotic property are allowed for submerged fermentation to produce crude extract which can be used in secondary screening.

### 2.6. Submerged-State Fermentation

For the production process, the submerged fermentation technique was used. Yeast malt extract broth (YMEB) was the primary medium in which the actinomycetes with higher zone of inhibition was kept with storage by continuous shaking for 4 days at 28°C. The extract was separated by centrifugation at 3000 rpm for 20 minutes. After the centrifugation, the pellet was discarded, and the supernatant was mixed with double the volume of the chilled acetone and left overnight [7]. The next day, the mixture was centrifuged at 5000 rpm for 20 minutes. Thus, the obtained pellet was mixed with ethyl acetate (76% w/v).

### 2.7. Secondary Screening (Agar Well Diffusion Method)

Mueller Hinton Agar (MHA) was used for the agar well diffusion method. Bacterial suspension of the ATCC culture of *Escherichia coli, Staphylococcus aureus*, *Klebsiella pneumoniae*, *Bacillus* spp., and *Pseudomonas aeruginosa* was compared with standard 0.5 McFarland solution which was equivalent to 106–108 CFU/ml. A cotton swab was used to make the carpet culture of the bacterial suspension on the MHA plate by squeezing the excess on the tube while continuously rotating during the carpet culture. Inoculated plates were allowed to diffuse in room temperature. A sterile 6 mm diameter cork borer was used to make well on the agar in which 100 *µ*L crude extract was carefully dispensed into each well. The plate was allowed to diffuse for 1–2 hours and then incubated at 37°C for 24 hours. After the proper incubation period, the zone of inhibition was measured and noted.

### 2.8. Characterization of *Actinomycetes*

The potential antibiotic-producing actinomycetes are characterized based on the microscopic observation and colony morphology on the media with color variation and texture of the colony varying from waxy and fuzzy to powdery. Furthermore, the color ranges from light yellow-orange to orange-red colonies for the *Micromonospora,* shiny blackish, fuzzy, and filamentous for the *Nocardia,* powdery white and gray to pinkish for the *Streptomyces* (Table 1).

## 3. Results

**Figure 2:**
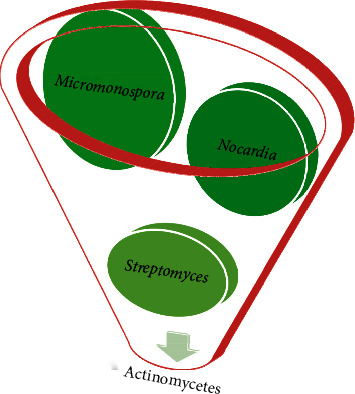
Graphical representation of different actinomycetes isolated. The graph represents the volumewise identification of actinomycetes species where the larger circle (*Micromonospora*) represents the higher identification in the sample followed by the medium circle (*Nocardia*) and small circle (*Streptomyces*).

**Figure 3:**
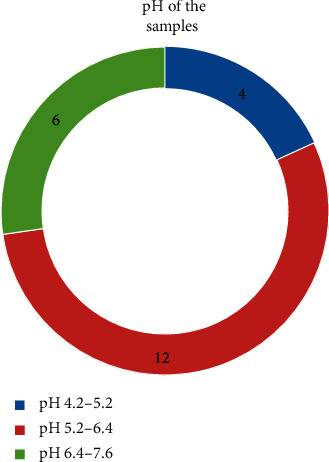
pH measurement of different samples. pH was measured using a pH meter. Among the isolated samples, 4 have the pH between 4.2 and 5.2, 12 have the pH between 5.2 and 6.4, and 6 have the pH between 6.4 and 7.6.

**Figure 4:**
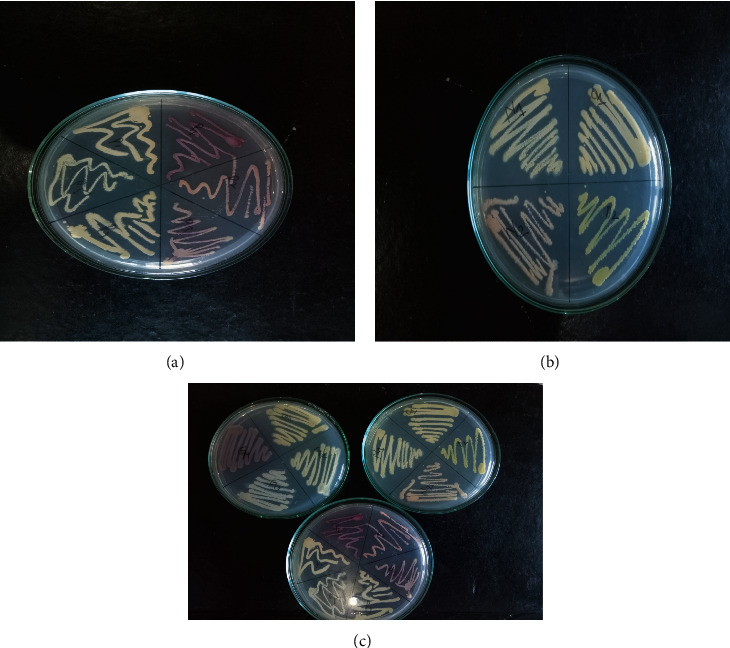
Subculture colony of actinomycetes. A Starch Casein Agar (SCA) plate showing the growth of actinomycetes with different colors and textures which can be utilized as macroscopic distinguishing characteristics (a), (b), and (c).

**Figure 5:**
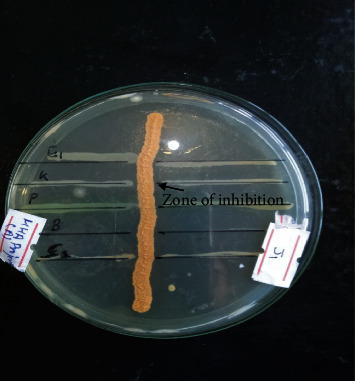
Primary screening of isolates. Subcultured isolates streaked line on the Mueller Hinton Agar (MHA) which was screened against different bacteria, (E) *E. coli*, (K) *Klebsiella pneumoniae*, (B) *Bacillus* species, (S) *Staphylococcus aureus,* and (P) *Pseudomonas aeruginosa* (as shown by the arrow).

**Figure 6:**
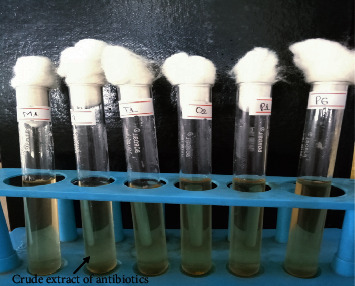
Crude extract of antibiotics. Test tube with the crude form of antibiotics following secondary submerged-state fermentation.

**Figure 7:**
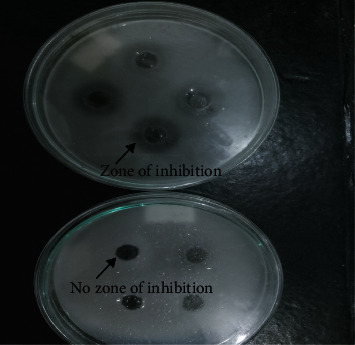
Secondary screening of isolates. Two plates showing the agar well diffusion method (top: the plate with zone of inhibition to bacterial lawn (as shown by arrow); below: the plate without zone of inhibition to bacterial lawn).

From the different isolates isolated, 12 isolates were found to be *Micromonospora*, 9 were *Nocardia*, and 7 were *Streptomyces* (Table 2 and Figure 2). The pH of the most samples was between 5 and 7 (Figure 3). This finding was solely based upon macroscopic colony characteristics on the SCA plate and microscopic characteristics (Figures 1 and 4). The perpendicular line streaking on the Mueller Hinton Agar (MHA) also showed the zone of inhibition against the perpendicularly streaked bacterial lawn (Figure 5). Eight isolates showed effective results against the tested bacteria (Table 3). Also, crude extract of antibiotics was extracted (Figure 6) and allowed for the well diffusion which showed a good zone of inhibition against the microorganism swabbed (Figure 7). From the isolates of primary screening, 4 isolates showed effective results against the tested bacteria (Table 4).

Out of a total of 22 samples, 47 colonies were isolated, among which 24% were used for subculturing and others showed contamination or no growth at all. Among these, only 17.02% were effective by perpendicular streaking with the ATCC culture, and among those, also only 8.52% showed the antibiotic property by the antibiotic produced through submerged-state fermentation technology (Tables 3 and 4). The significant number of crude forms extracted showed the zone of inhibition with the bacterial lawn used, indicating there is a high chance of good quality of antibiotics produced from them. Thus, we propose that if the process is standardized in large scale for purification of the crude form, cost-effective and good-quality antibiotics can be produced from the natural source.

## 4. Discussion

Twenty-two samples were used for the screening purpose, among which 17 samples were water samples and 5 were soil samples from different regions of Nepal varying in altitude. Among the samples, few did not show growth in the subculture. Furthermore, only those with macroscopic identification were used for the perpendicular streaking and for observing the zone of inhibition for determining the antibiotic property. Also, those showing an antibiotic property were applied for the antibiotic production process by extracting crude extract through the submerged fermentation process, and finally, its activity was checked by the well diffusion method.

The ATCC culture of *E. coli, P. aeruginosa*, and *S. aureus* was used for observing the antibiotic effect of actinomycetes, but the antibiotic effect was only observed for *E. coli* followed by a few cases in *S. aureus.* There were no any specific criteria for selection of the testing pathogens. *E. coli* is the most common cause of most water- and food-borne infections, and we wanted to check whether the antibiotic extracted would effectively act upon these infectious bacteria. We found that the crude form of the antibiotic extracted was effective against these opportunistic pathogens. We also cover the Gram-positive bacteria in our study. However, we found that most of the extracts are effective against Gram-negative bacteria rather than Gram-positive bacteria, indicating that the antibiotics extracted may not have the ability to cross the thick wall of peptidoglycan present in Gram-positive bacteria. The characteristics of antibiotics resemble the properties of the cephalosporins, fluoroquinolones, aminoglycosides, imipenem, and broad-spectrum penicillin with or without *β*-lactamase inhibitors. Also, more antibiotic properties were shown by the extract of water rather than of soil. Although most part of the country was not included on the research, regions from lower altitude to higher were included.

According to previous studies, soil was the higher source of *Actinomycetes*, showing an antibiotic effect on both Gram-positive and Gram-negative bacteria, but this research aided the water source beside the soil. According to Pramanik et al. [17], most of the isolates from the Sundarban grove ecosystem were found to be *Streptomyces*, and among the 54 isolates, only 9 showed higher antimicrobial properties against a wide range of bacteria along with phytopathogenic fungus [17]. In comparison to this result, our study revealed less isolates with higher antimicrobial property, but the result varied due to the difference in the topography difference of the sample site and procedure involved. Beside these, a wide range of areas have been covered on the research performed ranging from the height of 300m to the 1850m. Thus, it is possible to isolate actinomycetes even from higher altitude. Similarly, a study by Duddu and Guntku [1] also reported a similar result in which only one isolate among all examined actinomycetes isolates showed an effective antimicrobial activity against *E. coli*, *S. aureus*, *Bacillus pumilis*, and *Candida albicans*. Despite being the species of *Actinomycetes*, properties and characteristics at various levels of pH and temperature with climatic variation differ from each other. This was related by the Singh et al. [16] in their research paper. They found the highest biomass of actinomycetes were isolated from the incubation of 6–7 days on 28°C along with pH 7 during the extraction process. Although two factors, incubation days and temperature, were maintained accordingly in the research, lack of knowledge on the pH maintenance may have hindered the extraction process on our research.

Furthermore, the collection of the sample was based on the different sites such as moist soil under the tree, field soil, soil of water sediments, lake water, stringent water, pond, and stream in the research conducted. Unlike the study, Bizuye et al. [18] collected most soil samples from the rhizosphere, cooking house, cattle breeding area, house-waste disposal area, and industrial-waste disposal area. Also, soil was collected from very low depth up to the 11 cm in their study [18]. In comparison to the study by Bizuye et al. [18], we found that low or even nil number of isolates from the sample with low depth but significantly higher number from samples were collected from the higher depth region and from moist area. Thus, it can be assumed that, in comparison to the surface, actinomycetes prefer deeper habitation with the damp and moist condition. As most actinomycetes are able to hydrolyze urea, starch with resistance of 5% NaCl, and optimum temperature of 25–30°C according to Grebhoyness et al. [7], it can be predicted that most of these actinomycetes from soil are nonpathogenic as most pathogenic actinomycetes only grow at 37°C, which is the body temperature of human beings. Furthermore, resistance to 5% NaCl means they are not tolerant unlike marinophiles which can tolerate higher concentrates of salt. Along with these, hydrolysis of starch may be due to aminolytic actinomycetes and hydrolysis of urea may be due to the use of urea as a nitrogen source. These results may be interpreted from our cases as soil was used as the sample along with growth at 28°C; therefore, most isolates may also hydrolyze the starch and urea if tested. Although the number of *Streptomyces* was higher on their cases, we have a comparatively higher number of *Micromonospora.* Thus, higher number of novel antimicrobial compounds, namely, rifamycin, can be developed if proper extraction and purification process can be applied from the *Micromonospora.*

In Nepal, which is rich in topography and diversity with a wide range of geographical distribution, ecological variation may be seen with microorganisms' habitation; thus, exploration in this field might be very useful in drug discovery and designing. Different research studies till now have been based upon different location sites like the one where we performed. For example, Rai et al. [4] also isolated actinomycetes from the Hilly region covering the Kathmandu region as the location site; however, we covered from the Terai to Himalayan region in our study. Both studies showed that there is a wide possibility of determining the different strain of actinomycetes on the various part of Nepal. As conclusion of these results, higher research activities till the genotyping and phylogenetic analysis are highly recommended further by taking the different location of Nepal as the sample site.

Furthermore, use of antibiotics is widely spread in Nepal and poses a serious threat to the health and development of the country. Actual industrial antibiotic preparation has low sensitivity as stated by Gebreyonannes et al. [7], according to which crude extract extract from Lake Tana, Ethopia, was shown to be more sensitive than tetracycline. Also, the synthetic method is time consuming and requires expertise and highly equipped laboratory facilities. Extraction of antibiotics from crude extracts of a natural source will change the antibiotic to high sensitivity and specificity result.

Antibiotics therapy has been increasing day by day in Nepal. Due to higher cost, many people are being devoid of this therapy. Use of a natural source to extract antibiotics is a simple process, cost effective, less biohazardous to perform within limited space, and has good sensitivity along with specificity which is most desired in developing nations like Nepal in order to improve the health status of people and increase the GDP of the nation by decreasing the dependency with foreign nations for medicines. This study has the importance in direct manufacture of antibiotics which have less effect than the synthetically manufactured antibiotics. Also, this study can be valuable in decreasing the side effect of use of synthetic antibiotics and improving the sensitivity of antibiotics by decreasing the resistance property. Currently, many microorganisms show resistance to many antibiotics, and its ratio is increasing day by day to form MDR. This might be due to the modification of antibiotics during the manufacture process in the industry. The use of naturally isolated antibiotics would significantly reduce the MDR property of the microorganism, thereby providing safe antibiotic therapy against infection. This type of study and isolated antibiotics can be used for the treatment against a different type of infection at local level since it does not use most of the costly materials for its preparations. Furthermore, this study will help to increase the benefits of antibiotics therapy and improve the economic condition of nation which is essential to be utilized by the different governmental health sectors for maintaining the quality of life of people.

As this study was confined to few samples with few identification methods, it does not reveal the picture of whole identification process with confident finding. Therefore, this type of study should be carried out covering a wide geographical area along with an advance identification process to find out a precise result. Implementation of adequate sampling and higher fermentation process along with purification step using a specific buffer would be appreciable for the appropriate finding. In conclusion, it would be wise to use a natural source for the antibiotic production as it is cost effective and beneficial healthwise.

## 5. Conclusions

This study is a very useful advancing step on the field of diagnostic area. It isolated the antibiotics from the available natural source, thus being less reliable on the synthetic materials. Different antibiotics from different actinomycetes along with their strength of action against Gram-negative and Gram-positive bacteria were checked. The effectiveness of isolated antibiotics was also checked, but it could not be compared with synthetic; otherwise, it is expected to be similar or higher in some cases. The result is hoped to be positive with a better result from the isolated antibiotics. The finding of results would be beneficial for the respective area if utilized on the large scale for manufacturing antibiotics for reaching the hand of every individual including the poor one.

## Figures and Tables

**Table 1:** Identification of actinomycetal isolates based on morphological and cultural characteristics.

Colony characteristics on SCA	Microscopic characteristics	Actinomycetes isolated
Light yellow-orange to orange-red colonies, occasionally brown maroon or blue green, the dark brown to black spore colonies' surface darkens with spores	Fine substrate mycelium with spores like a cluster of grapes, no aerial mycelium	*Micromonospora*
The colony appears waxy and shiny; several millimeters in diameter; aerial filaments are formed; and the colony surface becomes dull and fuzzy	Gram positive, non-acid-fast, show pleomorphically ranging from a bacillary to coccoid structure; occasionally limited mycelium is found, which fragments readily to produce rod-shaped or coccoid cells	*Nocardia*
Powdery, colony appears convex, concave or flat surface; white gray to pinkish color colony	Filaments long highly branched and nonfragmented aerial filaments with spiral coil or multiple branching and long-chain spores	*Streptomyces*

**Table 2:** Sample site, sample size, antibiotic producer, and Genus classification.

Coding	Sample (water and soil)	Altitude	No. Of colonies isolated	Subculture with the antibiotic activity	Samples for fermentation	Genus (microscopic observation)
A	Sangapokhari, Sanga	1501m	3	A1, A3	A1	*Streptomyces, Micromonospora*
B	Tharapokhari, Banepa	1461m	1	—	—	*Micromonospora*
C	Dyapokhari, Banepa	1462m	—	—	—	—
D	Siddha pokhari, Bhaktapur	1336m	—	—	—	—
E	Bhajyapukhu, Bhaktapur	1327m	—	—	—	—
F	Bhitrikot pokhari1, Pyuthan	1540m	4	G1, G4	G4	*Micromonospor, Nocardia*
G	Bhitrikot pokhari2, Pyuthan	1547m	4	—	—	*Micromonospor, Nocardia, Streptomyces*
H	Unknown lake, Pyuthan	1165m	1	—	—	*Micromonospora*
I	Ratodada Lake, Pyuthan	968m	2	—	—	*Micromonospora*
J	Unknown lake, Baitadi	1375m	3	—	—	*Streptomyces, Nocardia*
K	Unknown lake, Kailali	340m	3	—	—	*Micromonospora, Nocardia*
L	Unknown lake, Pyangau, Kathmandu	1488m	2	M1, M2	—	*Micromonospora, Nocardia*
M	Buddha pokhari, Lalitpur	1463m	2	—	—	*Micromonospora*
N	Siddha pokhari, Dhulikhel	1543m	—	—	—	—
O	Unknown lake, Shorakhutte, Kathmandu	1483m	2	—	—	*Micromonospora*
P	Unknown lake, Matchhegaun, Kathmandu	1424m	—	—	—	—
Q	Soil, Baitadi	1370m	6	P6	P6	*Micromonospora Streptomyces*
R	Brown soil, Chitlang	1757m	5	—	—	*Streptomyces, Nocardia*
S	Black wet soil, Chitlang	1754m	2	—	—	*Micromonospora, Nocardia*
T	Brown wet soil, Chitlang	1750m	4	T4	T4	*Streptomyces, Nocardia*
U	Light-brown soil, Chitlang	1853m	3	—	—	*Streptomyces, Nocardia*
V	Light-black soil, Chitlang	1755m	—	—	—	—

**Table 3:** Primary screening of the different isolates with effective results.

Isolates identified (spp.)	Sample number	Zone of inhibition (mm)				
		*Staphylococcus aureus* (ATCC 25923)	E.*coli* (ATCC 25923)	*Klebsiella pneumoniae* (ATCC 700603)	*Pseudomonas aeruginosa* (ATCC 27853)	*Bacillus* spp.
*Streptomyces*	A1	0	12	2	0	0
*Streptomyces*	T4	16	1	0	0	1
*Micromonospora*	P6	4	16	1	1	1
*Nocardia*	G4	17	4	12	1	0
*Streptomyces*	G1	2	2	3	0	0
*Streptomyces*	T4	0	12	8	0	0
*Streptomyces*	P6	0	3	0	2	5
*Micromonospora*	G4	0	14	0	0	4

**Table 4:** Secondary screening of the different isolates with effective results.

Isolates identified (spp.)	Sample number	Zone of inhibition (mm)				
		*Staphylococcus aureus* (ATCC 25923)	E.*coli* (ATCC 25923)	*Klebsiella pneumoniae* (ATCC 700603)	*Pseudomonas aeruginosa* (ATCC 27853)	*Bacillus* spp.
*Streptomyces*	A1	2	13	2	0	0
*Streptomyces*	T4	18	2	4	3	2
*Micromonospora*	P6	4	12	2	2	1
*Nocardia*	G4	18	4	10	1	2

## Data Availability

Data used in this work can be obtained on request to the corresponding author.
